# Growing old before growing rich: inequality in health service utilization among the mid-aged and elderly in Gansu and Zhejiang Provinces, China

**DOI:** 10.1186/1472-6963-12-302

**Published:** 2012-09-04

**Authors:** Yang Wang, Jian Wang, Elizabeth Maitland, Yaohui Zhao, Stephen Nicholas, Mingshan Lu

**Affiliations:** 1 Center for Health Management and Policy, Shandong University, Shandong, China; 2 Australian School of Business, University of New South Wales and School of Business, Nanjing University, Nanjing, China; 3 China Center for Economic Research, Peking University, Beijing, China; 4 University of Newcastle, Australia and Guangdong University of Foreign Studies, Guangdong, China; 5 Department of Economics, University of Calgary and the Hong Kong University of Science & Technology, Calgary, Canada

**Keywords:** Aging, Inequality, Health service utilization, China

## Abstract

**Background:**

China’s recent growth in income has been unequally distributed, resulting in an unusually rapid retreat from relative income equality, which has impacted negatively on health services access. There exists a significant gap between health care utilization in rural and urban areas and inequality in health care access due to differences in socioeconomic status is increasing. We investigate inequality in service utilization among the mid-aged and elderly, with a special attention of health insurance.

**Methods:**

This paper measures the income-related inequality and horizontal inequity in inpatient and outpatient health care utilization among the mid-aged and elderly in two provinces of China. The data for this study come from the pilot survey of the China Health and Retirement Longitudinal Study in Gansu and Zhejiang. Concentration Index (CI) and its decomposition approach were deployed to reflect inequality degree and explore the source of these inequalities.

**Results:**

There is a pro-rich inequality in the probability of receiving health service utilization in Gansu (CI outpatient = 0.067; CI inpatient = 0.011) and outpatient for Zhejiang (CI = 0.016), but a pro-poor inequality in inpatient utilization in Zhejiang (CI = −0.090). All the Horizontal Inequity Indices (HI) are positive. Income was the dominant factor in health care utilization for out-patient in Gansu (40.3 percent) and Zhejiang (55.5 percent). The non-need factors’ contribution to inequity in Gansu and Zhejiang outpatient care had the same pattern across the two provinces, with the factors evenly split between pro-rich and pro-poor biases. The insurance schemes were strongly pro-rich, except New Cooperative Medical Scheme (NCMS) in Zhejiang.

**Conclusions:**

For the middle-aged and elderly, there is a strong pro-rich inequality of health care utilization in both provinces. Income was the most important factor in outpatient care in both provinces, but access to inpatient care was driven by a mix of income, need and non-need factors that significantly differed across and within the two provinces. These differences were the result of different levels of health care provision, different out-of-pocket expenses for health care and different access to and coverage of health insurance for rural and urban families. To address health care utilization inequality, China will need to reduce the unequal distribution of income and expand the coverage of its health insurance schemes.

## Background

Since Deng Xiaoping’s 1978 ‘Southern Journey’, China’s average growth rate of 10 percent has gained China middle-income status and moved 500 million people out of poverty. China now faces the challenge of breaking through the middle-income country trap, where countries attain middle-income status, but fail to go on to high-income status [1,2]. This challenge is significant. Today, 204 million remain in poverty, and China’s per capita income is projected to be only $US16000 in 2030 [3]. As China tackles the middle-income trap challenge, it will also be faced with managing a significant demographic transition. The old-age dependency ratio—defined as the ratio of those aged 65 and over to those between the ages of 15 and 64—will double over the next 20 years, while the working-age population will start to decline after 2015 [4-6]. By 2050, the elderly in China will account for 27 percent of the population. While this change in the age profile of the population will be accompanied by a decline in communicable diseases, an increasing number of elderly will raise the incidence of non-communicable diseases, placing new burdens on health care provision into the future. In short, China will grow old, before it grows rich, which will have major implications for health care provision [7].

A society should grant its citizens equal access to health care for equal need [8-10]. During the reform period, China moved away from the ideal of equal access for equal need, as the Government reduced its responsibility for state-owned health facilities and emphasized their autonomy. The shift to profit-oriented hospitals saw the prices of health care rise [11]; the average number of days in hospital increase to twice the OECD average; and an estimated third to half of all admissions to hospitals in China being unnecessary [4]. At the same time, the proportion of the population covered by health insurance dropped significantly. Finally, China’s recent growth has been unequally distributed, resulting in an unusually rapid retreat from relative income equality, which has impacted negatively on health services access, especially for the poor [12]. With a population of 1.3 billion, which is aging, and the negative effects of economic reform and unequal income distribution, China’s current and projected demand for health services is daunting. Amidst these challenges, significant variations within China’s aging population are predicted, with rural seniors and females having lower hospital utilization rates, but a higher utilization for physician visits, than their urban counterparts [13].

This paper measures the income-related inequality and horizontal inequity in inpatient and outpatient health care utilization among the mid-aged and elderly in two provinces of China. The two provinces selected comprise one urban developed province, Zhejiang, and one rural backward province, Gansu, which allows comparisons of health care utilization from two provinces experiencing different growth trajectories. In 2007, Zhejiang had a yearly per capita income of 11235RMB, the third highest among 31 provinces, while Gansu’s per capita income was only 4870RMB, ranking number 30 among 31 provinces [14]. The paper also analyzes data on health insurance to assess the impact of the various health insurance policy reforms on health service utilization. Finally, the paper provides a number of policy implications and suggestions.

In China’s pre-reform collectivist economy, the Rural Cooperative Medical System (RCMS) provided a needs-based insurance safety net for agricultural workers. With the emergence after 1978 of the Household Responsibility System, which shifted production from a collective to an individual family production system, the RCMS collapsed, leaving rural areas virtually without a health insurance system, while in non-rural areas, the Urban Employee Basic Medical Insurance (UEBMI) scheme was directly tied to formal employment status, which excluded over 400 million family members and unemployed and migrant workers [15,16].

Beginning in 2002, the Government implemented two schemes to increase health insurance coverage for the population. In rural areas, the New Cooperative Medical Scheme (NCMS) was piloted before being expanded nationally [15]. With the aim of universal urban health care coverage, the voluntary household-based Urban Resident Basic Medical Insurance (URBMI) scheme extended coverage to the unemployed, students, children, and the elderly. Our study focuses specifically on health insurance and healthcare utilization patterns, since studies have shown that health insurance is a key variable in ensuring health utilization equity, especially for the poor [17-21]. We expect that the health insurance reforms will have a positive impact on access to health care for middle and older aged Chinese.

## Methods

### Data and variables

The China Health and Retirement Longitudinal Study (CHARLS) is a large-scale survey targeting population aged 45 and above in China, providing a wide range of demography, socioeconomic status and health condition variables. All data, stripped of private identifying information, are freely available for research use (http://charls.ccer.edu.cn/charls/). The authors participated in the CHARLS questionnaire design. In this paper, health care utilization data from the pilot survey of CHARLS implemented in Gansu and Zhejiang in 2008 are analyzed. The probabilities proportional to size (PPS) sampling method was adopted [22], collecting data from 48 communities or villages in 16 counties or districts covering 2,555 individuals living in 1,570 households. Weights were applied to reflect the population structure of each province. We excluded observations with missing values in our analysis, leaving 1198 observations in Gansu and 1357 observations in Zhejiang.

Health care utilization was measured by inpatient and outpatient services. Outpatient utilization was defined as visiting, but not being admitted to a general hospital, specialized hospital, Chinese medicine hospital, community healthcare center, township hospital, health care post or village clinic/private clinic in the last 4 weeks for treatment. Inpatient utilization was defined as admission within the last year to medical facilities, including general hospital, specialized hospital, Chinese medicine hospital, community healthcare center, or township healthcare clinic.

The independent variables are described in Table 1 and include data on age (45- years; 55- years and; 65 years or above), gender, a five point self-rated health scale (from below 2 points for poor health to 4–5 points for good health), diagnosed disease status (including chronic, cardiovascular, digestive and cancer), education (less than six years; 7–12 years and greater than 12 years) and occupation (farmer or no job and employed, including self-employed). In rural areas of China it is difficult to distinguish the “farmer” and “no job” categories, which is also reflected in the CHARLS data. Since, no job accounted for a very small proportion of the total occupations, we combined “farmer” and “no job” into a single occupational category. Income quartiles data were collected, with the range of each quartile presented in Table 2, which clearly displays the relative socioeconomic standing of the two provinces. Data were also collected on residency (urban, town, countryside and special areas, such as special economic zones) and health insurance (NCMS, UEBMI and URBMI and Other, such as commercial insurance and free medical schemes).

**Table 1:** Mean and Concentration Indices of health service determinants in Gansu and Zhejiang

		**Gansu**		**Zhejiang**	
		**proportion**	**CI**	**proportion**	**CI**
45- male	1 if age greater than 45 (including 45) and less than 55 and male, 0 otherwise	0.186	0.057	0.17	0.253
55- male	1 if age greater than 55 (including 55) and less than 65 and male, 0 otherwise	0.153	0.019	0.167	0.053
65 or above- male	1 if age above 65 (including 65) and male, 0 otherwise	0.171	−0.157	0.169	−0.341
45- female	1 if age greater than 45 (including 45) and less than 55 and female, 0 otherwise	0.178	0.226	0.183	0.344
55- female	1 if age greater than 45 (including 55) and less than 55 and female, 0 otherwise	0.160	−0.049	0.167	−0.132
65 or above- female	1 if age above 65 (including 65) and female, 0 otherwise	0.152	−0.224	0.144	−0.410
Below 2 point	1 if self-rate health below 2 point, 0 otherwise	0.064	0.095	0.166	0.060
2- point	1 if self-rated health greater than 2 (including 2) and less than 3 point, 0 otherwise	0.303	−0.061	0.352	0.030
3- point	1 if self-rated health greater than 3 (including 3) and less than 4 point, 0 otherwise	0.372	0.078	0.349	−0.017
4-5 point	1 if self-rated health between 4 and 5 point (including 4 and 5), 0 otherwise	0.261	−0.121	0.133	−0.293
No disease	1 if not diagnosed as a disease (including chronic, cardiovascular, digestive disease or cancer) 0 otherwise	0.323	−0.028	0.384	0.018
Disease	1 if diagnosed as a disease (including chronic, cardiovascular, digestive disease or cancer) 0 otherwise	0.677	−0.009	0.616	−0.051
0 < =edu < = 6	1 if receiving 0–6 years’ education, 0 otherwise	0.718	−0.090	0.766	−0.087
6 < edu < =12	1 if receiving 7–12 years’ education, 0 otherwise	0.261	0.177	0.206	0.228
12 < edu < =18	1 if receiving 13–18 years’ education, 0 otherwise	0.021	0.167	0.028	−0.171
UEBMI	1 if having insurance for urban employees,0 otherwise	0.119	0.230	0.138	0.205
URBMI	1 if having insurance for urban residents,0 otherwise	0.057	0.536	0.027	0.281
NCMS	1 if having NCMS, 0 otherwise	0.720	−0.122	0.713	−0.062
Other insurance	1 if other, 0 otherwise	0.112	0.063	0.122	−0.132
Farmer or no job	1 if farmer or no job, 0 otherwise	0.887	−0.076	0.628	−0.181
Employed or self	If having a job or running a business, 0 otherwise	0.113	0.467	0.372	0.240
quartile1	1 If in income quartile 1 (lowest), 0 otherwise	0.234	−0.753	0.234	−0.760
quartile2	1 if in income quartile 2, 0 otherwise	0.243	−0.249	0.246	−0.276
quartile3	1 if in income quartile 3, 0 otherwise	0.258	0.189	0.249	0.221
quartile4	1 if in income quartile 4 (highest), 0 otherwise	0.265	0.653	0.271	0.614
Urban area	1 if residing in urban area, 0 otherwise	0.142	0.309	0.085	−0.098
Town	1 if residing in town area, 0 otherwise	0.136	0.110	0.173	0.266
Country	1 if residing in country area, 0 otherwise	0.629	−0.132	0.452	−0.193
Other residence	1 If residing in special area where nonfarm employment constitutes at least 70% of the work force, such as special zone, state-owned farm enterprise etc., 0 otherwise	0.093	0.098	0.29	0.087
SAMPLE SIZE		1198		1357	

**Table 2:** Range of Income Quartiles

	**Gansu**		**Zhejiang**	
	**Min**	**Max**	**Min**	**Max**
Quartile 1	−7278	420	−18000	2500
Quartile 2	433	1590	2533	7287
Quartile 3	1616	4985	7300	15000
Quartile 4	5000	147301	15000	358400

### Methodology

The paper employs the standard concentration index (CI) developed by Wagstaff et al. to quantify and compare the degree of income related inequality in the health utilization variables and the horizontal index (HI) to measure need-standardized health care utilization [23-25]. Following Wagstaff et al. (2008), the CI is defined as twice the area under the concentration curve and the 45 degree equality line, where a concentration curve plots the cumulative percentage of the health variable (y-axis) against the cumulative percentage of the population, ranked by income, beginning with the poorest, and ending with the richest (x-axis). If the concentration curve lies on the 45 degree line, then there is perfect income equality in the health variable. CI can be written:

(1) $$\text{C=}\frac{2}{\text{nμ}}\sum_{\text{i=}1}^{\text{n}}{\text{h}}_{\text{i}}{\text{R}}_{\text{i}}-1$$

Where *hi* is the health sector variable, μ is its mean, and *Ri*is the fractional rank of individual *i* in the distribution of income, with *i* = 1 for the poorest and *i* = N for the richest.

The CI ranges from −1 to +1, where CI is equal to zero for equality in health, a negative value when the curve lies above the line of equality, indicating disproportionate concentration of the health variable among the poor, and a positive value when it lies below the line of equality care. Of course, CI equal to zero might reflect perfect equality or the concentration curve crosses and re-crosses the 45 degree line where the above and below the line areas exactly cancel each other. When the health variable is a “bad” such as visits to outpatient or inpatient facilities, a negative value of the concentration index means ill health is higher among the poor. A larger absolute negative value of CI indicates more pronounced inequality.

The concentration index decomposing method reveals the sources of income-related inequalities. Taking health care use as the dependent variable, it is defined in the following linear model:

(2) $${\text{h}}_{\text{i}}\text{=δ+}\sum_{\text{k}}{\text{γ}}_{\text{k}}{\text{x}}_{\text{ik}}+\sum_{\text{p}}{\text{γ}}_{\text{p}}{\text{z}}_{\text{ip}}{\text{+ϵ}}_{\text{i}}$$

where two types of explanatory variables are identified: need variables (${}^{x}k$), such as age, gender, self-assessed health and disease history, and non-need variables (${}^{z}p$) including insurance status, education, occupation and residency location. δ, y and E denote a constant, coefficient and error term respectively.

The concentration index for health care utilization can be decomposed as:

(3) $$\text{c=}\sum_{\text{k}}\left(\frac{{\text{y}}_{\text{k}}\bar{{\text{x}}_{\text{k}}}}{\text{μ}}\right){\text{C}}_{\text{k}}+\sum_{\text{p}}\left(\frac{{\text{y}}_{\text{p}}\bar{{\text{z}}_{\text{p}}}}{\text{μ}}\right){\text{c}}_{\text{p}}+\frac{{\text{GC}}_{\text{δ}}}{\text{μ}}$$

Where $C_{k}$ and $C_{p}$ represent need and non-need variables’ concentration indices, and $GC_{\delta }$ is error term of health care’s CI. The summations are considered as contributions of variables. While variables in health research, such as hospital visits, are frequently non-linear or skewed, Van Doorslaer et al. (2008) have shown that the measurement of horizontal inequality is not sensitive to the specification. The paper employs logit regression models [26].

The horizontal inequity index (HI) is calculated to gauge the influence of social disparity after standardizing for different needs, which are proxied by gender, age, self-assessed health and chronic disease history. HI is computed as a standardization approach by subtracting the need-related inequality from the total so as to identify the pure effect of socioeconomic status: in other words, it measures inequity. HI with a positive (negative) value indicates pro-rich (pro-poor) inequity just as CI. The formula is written as:

(4) $$\text{HI=C}\sum_{\text{k}}\left(\frac{{\text{y}}_{\text{k}}\bar{{\text{x}}_{\text{k}}}}{\text{μ}}\right){\text{C}}_{\text{k}}$$

## Results

### Distribution of health care utilization and its determinants

The means and concentration indices of health service utilization and its determinants are presented in Table 1 and 3. All the values were weighted by the sampling probability. The proportion of interviewees reporting having received outpatient and inpatient service in Gansu (outpatient 17.1% and 7.7% inpatient) was comparable to those in Zhejiang (outpatient 15.7% and 6.0%). The concentration indices in Table 3 reveal a pro-rich inequality in the probability of receiving health service utilization in Gansu (CI outpatient = 0.067; CI inpatient = 0.011) and outpatient for Zhejiang (CI = 0.016), but a pro-poor inequality in inpatient utilization in Zhejiang (CI = −0.090).

**Table 3:** Measure of Health Care Utilization Inequality in Gansu and Zhejiang

	**Probability**	**CI**	**HI**
Gansu Outpatient	0.177	0.06669	0.04626
Gansu Inpatient	0.071	0.01082	0.03911
Zhejiang Outpatient	0.157	0.01612	0.04858
Zhejiang Inpatient	0.060	−0.09001	0.03849

The means for categories of determinants in Table 1 show proportionate distributions of the respondents across those categories, where the categories sum to one. Age and gender shared a similar distribution among the population in Gansu and Zhejiang and the CIs demonstrate that the poor were concentrated among the old-aged (65 or above male: CI Gansu = −0.157; CI Zhejiang = −0.341), especially the female (65 or above female: CI Gansu = −0.224; CI Zhejiang = −0.410), subpopulations. We speculate that women were less likely than men to be financially independent, especially at older ages. Second, the CIs for the older aged groups were significantly higher in prosperous Zhejiang than rurally backward Gansu. This result may reflect the Chinese tradition in rural areas for old aged parents to live in extended households as opposed to more urban and prosperous Zhejiang where aged, and likely poor parents, lived by themselves.

For self-assessed health status, there was a pro-poor bias in reporting good health (4–5 point: CI Gansu = −0.121; CI Zhejiang = −0.293) and a pro-rich bias in reporting bad health (CI Gansu = 0.095; CI Zhejiang = 0.060). This self-reporting on health runs counter both to expectations that the poor have poorer health, and the data on diagnosed disease where the poor in both provinces were more likely to be diagnosed as suffering from disease (Disease: CI Gansu = −0.009 and CI Zhejiang = −0.051). We comment on possible biases in self-reporting below. High socioeconomic status variables, such as education and type of employment, proxied high income in both provinces, so that the poor in Table 1 were concentrated in the groups with lower education and the worst jobs. There was one anomaly: in Zhejiang (CI = − 0.171), individuals with college education had a lower income. We speculate that most of the rich are small business operators and they do not generally have a college education. Farmer or those with no job accounted for 89 percent of the sample in Gansu and 63 percent in Zhejiang, and both groups were disadvantaged (Farmer/no job: CI Gansu = −0.076; CI Zhejiang = −0.181), while employed or self employed groups were socioeconomically well off (Employed/self-employed: CI Gansu = 0.467 and CI Zhejiang = 0.240).

Concentration indices for geographical areas clearly demonstrate the relatively wealthy and less well-off areas within each province. For Gansu, 63 percent lived in the country, and they were poor (CI = −0.132) as were the 45 percent of Zhejiang’s country respondents (CI = −0.193). But 8.5 percent of the Zhejiang respondents living in urban areas (CI = −0.098) were also poor, an indication of the level of disadvantage faced by urban workers even in one of China’s most prosperous provinces. We argue that in a rich province like Zhejiang, the richest part of the population did not choose to live in the center of the city, but rather in the suburbs that is proxied by the town variable.

Insurance schemes had a pro-rich impact, except for NCMS, which was negative for both Gansu (CI = −0.122) and Zhejiang (CI = −0.062), indicating that the poor were concentrated in the NCMS scheme in both provinces. Across the socioeconomic and health variables where the poor were concentrated, such as the elderly and older females, low education, living in rural areas and farmer or no employment, the CIs were higher in Zhejiang than Gansu. There is a comparable story for the rich, who were more concentrated in the pro-rich variables in Zhejiang than in Gansu (see Table 1). There is an interesting anomaly in Chinese economic development that the disparities in the rich province were greater than the disparities in the poor province, across a range of socioeconomic and health measures.

### Decomposition of inequality

**Figure 1:**
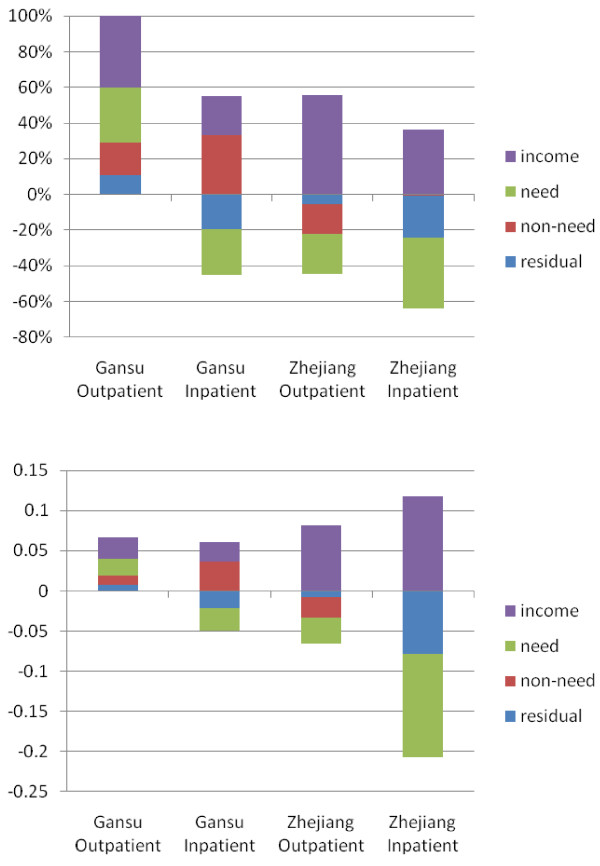
**Decomposition of Health Care Utilization Inequality in Gansu and Zhejiang.** Note: Upper one represents contribution in value; lower one represents contribution in percentage (or absolute value of each contribution divided by the summation of all four absolute values).

We also analyzed horizontal inequity in health care utilization, where we control income inequality for need and non-need variation and a residual. In summary Table 3, all HIs are positive (HI Gansu Outpatient: 0.046, Inpatient: 0.039; Zhejiang Outpatient: 0.049, Inpatient: 0.038), which indicates a pro-rich bias in inpatient utilization and outpatient utilization in both provinces. Table 4 and Figure 1 reveal the contribution of the income, need and non-need variables and the residual factor to outpatient and inpatient equity by value and percentage. In Figure 1, the sum of the bars would be zero if utilization had been equal across all income groups, and the need bar would be the only bar to appear. Our data in Figure 1 reveal substantial inequities in health care utilization.

**Table 4:** Decomposition of Health Care Utilization Inequality in Gansu and Zhejiang

	**income**	**need**	**non-need**	**residual**
Gansu Outpatient	0.02688	0.02043	0.01223	0.00716
	40.30%	30.63%	18.34%	10.73%
Gansu Inpatient	0.02409	−0.02829	0.03637	−0.02135
	21.88%	25.69%	33.03%	19.39%
Zhejiang Outpatient	0.08129	−0.03246	−0.02507	−0.00765
	55.50%	22.16%	17.12%	5.22%
Zhejiang Inpatient	0.11736	−0.1285	0.00002	−0.07887
	36.14%	39.57%	0.01%	24.29%

What stand out in Figure 1 and Table 4 are the differences in the role of income, need and non-need across the two provinces and across the utilization of different health care services. Income was the dominant factor in health care utilization for outpatients in Gansu (40.3 percent) and Zhejiang (55.5 percent); however, for inpatient services, income (36.1 percent) was the second most important factor in Zhejiang, behind need (39.6 percent), and the third most important factor in Gansu (income = 21.9 percent), behind non-need (33.0 percent) and need (25.7 percent). For outpatient care, income substantially affected whether a person sought medical advice to a greater extent than a person’s decision to seek inpatient care.

To investigate the impacts of need and non-need, we investigate each factor’s contribution, where a factor contributes to inequity in health care use if it is both distributed unequally by income and has an effect on the probability of using health care. Contribution is not meant in a causal sense, but rather it helps to explain the association between use and income rank through its partial association with utilization of care facilities [26].

Table 5 presents the marginal effects and their contribution to the CI. It can be seen that the contributions to the needs variables, especially 65 or above males, 55- and 65 or above women, self-evaluated poor health and disease, tend to be negative, with inpatient and outpatient care concentrated in the poor. This is consistent with the poor suffering more health problems, and suffering them more severely than the rich.

**Table 5:** Contributions of Determinants to Concentration Indices (absolute value and percentage)

	**Gansu**								**Zhejiang**							
	**outpatient**				**inpatient**				**outpatient**				**inpatient**			
	**Marg**	**P val.**	**Contri**	**Prop**	**Marg**	**P val.**	**Contri**	**Prop**	**Marg**	**P val.**	**Contri**	**Prop**	**Marg**	**P val.**	**Contri**	**Prop**
55- male	0.032	0.482	0.00053	0.79%	0.051	0.217	0.00212	19.59%	0.101	0.082	0.00564	34.99%	0.061	0.149	0.00892	−9.91%
65 or above- male	0.016	0.719	−0.00242	−3.63%	0.074	0.138	−0.02793	−258.13%	0.012	0.800	−0.00441	−27.36%	0.066	0.180	−0.06341	70.45%
45- female	0.154	0.005	0.03498	52.45%	0.023	0.471	0.01302	120.33%	0.042	0.370	0.01682	104.34%	0.001	0.961	0.00105	−1.17%
55- female	0.112	0.052	−0.00493	−7.39%	0.02	0.534	−0.00220	−20.33%	0.054	0.292	−0.00761	−47.21%	0.002	0.951	−0.00074	0.82%
65 or above- female	0.052	0.323	−0.01002	−15.02%	0.012	0.708	−0.00576	−53.23%	0.039	0.469	−0.01467	−91.00%	0.062	0.255	−0.06101	67.78%
Below 2 point	−0.098	0.000	−0.00338	−5.07%	−0.028	0.030	−0.00241	−22.27%	−0.120	0.000	−0.00757	−46.96%	−0.051	0.000	−0.00842	9.35%
2- point	−0.169	0.000	0.01778	26.66%	−0.06	0.000	0.01573	145.38%	−0.130	0.000	−0.00884	−54.84%	−0.045	0.001	−0.00800	8.89%
3- point	−0.054	0.010	−0.00886	−13.29%	−0.046	0.000	−0.01881	−173.84%	−0.061	0.011	0.00229	14.21%	−0.037	0.006	0.00363	−4.03%
Disease	0.095	0.000	−0.00325	−4.87%	0.024	0.071	−0.00205	−18.95%	0.071	0.001	−0.01411	−87.53%	0.001	0.959	−0.00052	0.58%
			0.02043	30.63%			−0.02829	−261.46%			−0.03246	−201.36%			−0.12850	142.76%
0 < =edu < = 6	0.077	0.385	−0.02819	−42.27%	0.019	0.601	−0.01734	−160.26%	0.093	0.107	−0.03932	−243.92%	0.016	0.678	−0.01770	19.66%
6 < edu < =12	0.155	0.328	0.04055	60.80%	−0.001	0.987	−0.00065	−6.01%	0.059	0.587	0.01763	109.37%	0.038	0.598	0.02971	−33.01%
UEBMI	0.012	0.822	0.00186	2.79%	0.057	0.307	0.02199	203.23%	0.061	0.301	0.01101	68.30%	0.039	0.387	0.01842	−20.46%
URBMI	0.021	0.711	0.00362	5.43%	0.014	0.706	0.00602	55.64%	0.123	0.319	0.00594	36.85%	0.072	0.382	0.00909	−10.10%
NCMS	−0.068	0.126	0.03378	50.65%	−0.010	0.613	0.01238	114.42%	0.020	0.517	−0.00561	−34.80%	0.032	0.072	−0.02348	26.09%
Employed or self	−0.013	0.704	−0.00388	−5.82%	−0.004	0.841	−0.00297	−27.45%	−0.027	0.234	−0.01535	−95.22%	0.005	0.766	0.00744	−8.27%
Urban area	−0.023	0.622	−0.00571	−8.56%	−0.034	0.014	−0.02104	−194.45%	0.168	0.05	−0.00894	−55.46%	0.044	0.344	−0.00612	6.80%
Town	0.158	0.038	0.01333	19.99%	0.025	0.413	0.00526	48.61%	0.080	0.073	0.02344	145.41%	0.002	0.935	0.00153	−1.70%
Country	0.092	0.021	−0.04313	−64.67%	−0.028	0.196	0.03272	302.40%	0.025	0.308	−0.01387	−86.04%	0.013	0.368	−0.01887	20.96%
			0.01223	18.34%			0.03637	336.14%			−0.02507	−155.52%			0.00002	−0.02%
quartile1	−0.026	0.421	0.02590	38.84%	0.003	0.867	−0.00745	−68.85%	−0.063	0.025	0.07138	442.80%	−0.034	0.026	0.10080	−111.99%
quartile2	−0.023	0.463	0.00787	11.80%	−0.008	0.659	0.00682	63.03%	−0.014	0.634	0.00605	37.53%	−0.039	0.009	0.04412	−49.02%
quartile3	−0.025	0.386	−0.00689	−10.33%	0.036	0.115	0.02472	228.47%	0.011	0.730	0.00386	23.95%	−0.030	0.018	−0.02756	30.62%
			0.02688	40.31%			0.02409	222.64%			0.08129	504.28%			0.11736	−130.39%
CI			0.06669	100.00%			0.01082	100.00%			0.01612	100.00%			−0.09001	100.00%
Contribution			0.05934	88.98%			0.03208	296.49%			0.02376	147.39%			−0.01112	12.35%
Residue			0.00716	10.74%			−0.02135	−197.32%			−0.00765	−47.46%			−0.07887	87.62%

Prop is contribution divided by the each outpatient or inpatient CI. Some powerfully influential variables could exceed 100% (contribution is bigger than overall inpatient or outpatient CI.)

The non-need contribution to inequity in Gansu and Zhejiang outpatient care had the same pattern across the two provinces, with the factors evenly split between pro-rich and pro-poor biases. Those with the lowest education had a pro-poor bias on the use of health services and the better education a pro-rich bias. Employed or self-employ, urban and country residence had a pro-poor outpatient service utilization bias in both provinces. The insurance schemes were strongly pro-rich, except NCMS in Zhejiang, which was pro-poor. As noted above, data from other countries generally show a pro-poor bias in universal health care insurance schemes, like NCMS, but this was not the case for the universal, but voluntary, URBMI or NCMS in Gansu.

While the non-need factors displayed the same biases, except NCMS, for outpatient utilization in the two provinces, this was not the case for inpatient utilization. Inpatient utilization shared the same pro-poor bias for lowest education and urban and the same pro-rich bias for town and URBMI and UEBMI insurance, but different pro-rich or pro-poor biases for all other factors. Again, Zhejiang’s NCMS had a pro-poor bias, but NCMS for Gansu was pro-rich.

## Discussion and conclusion

There was pro-rich inequality in the probability of receiving health service utilization except for inpatient in Zhejiang (negative CI), where inpatient service utilization was more concentrated among the poor. After controlling for need variation, all HIs also favored the financially wealthy in both provinces. Our results reveal the complexity of factors accounting for outpatient and inpatient utilization and the differences between provinces. While the poor were of poorer health, income was the dominant factor shaping access to outpatient care, but not inpatient care. It was not simply the case that increasing per capita income was making it easier for everyone to access increased medical care. Regional disparities and differential health service provision had a pervasive impact on health care access. For example people in different provinces at similar income levels had different accessibility to health services due to the management of health facilities at the regional-local level. These intra-provincial differences compounded provincial level differences, especially the ability of local governments in economically-developed regions to build health facilities compared to poor provinces, where health facilities are both more limited and, in agricultural provinces, more dispersed [27].

Our results also highlight the differences between inpatient and outpatient care. Generally, poorer people are inclined to self-treat rather than to seek inpatient services and our data show that the poor are concentrated in rural areas, with relatively poor access to medical facilities. Inpatients are more likely to face severe disease problems, but the expense (with the tendency of families to forego medical treatment due to costs) and the availability of hospitals, constrained inpatient treatment. Besides self-treatment, it is likely the poor access outpatient services for a range of treatments for which the rich seek inpatient care, which means that our data probably underestimate the pro-rich bias in access to inpatient health service care.

The effect of different health insurance schemes differed according to their financing regimes, which impacted on the accessibility of health services. NCMS, for example, is underwritten by both the central and provincial governments, but the county-unit governments have most of the responsibility for setting parameters of the program, such as user fees or, equivalently, insurance premiums, resulting in large differences across counties in health service coverage and the percentage of total costs reimbursed. The different effects of NCMS between Gansu and Zhejiang, and also the pro-rich bias of the other health insurance schemes, were due to different contribution amounts and differences in health insurance coverage from province to province and region to region. For both provinces, the level of insurance premiums excluded a subpopulation of poor people from health coverage [4].

These insurance results are broadly consistent with the existing literature on the impact of China’s health insurance on health care utilization. In a study of pre and post-urban health insurance reform in China, Liu et al. found health insurance reform improved access to essential health care [28]. For women and migrant workers, recent research suggests that whether an individual takes advantage of health service after illness is determined by insurance status, household income and education level [29-31]. In a study comparing 2003 and 2008 Chinese National Health Services Survey (CNHS) data on rural workers, Zhou et al. found a pro-rich inequity in rural inpatient utilization, but concluded that greater access to health insurance and rising incomes for both the rich and poor would reduce, but not close, the inequality gap between rich and poor [32].

It is illustrative to compare the Chinese health insurance impact on health access with the experiences in other countries. For Taiwan, Hong Kong and South Korean, there was a tendency towards ‘equal treatment for equal need’, except general practitioner and dental visits in Hong Kong [33]. In Australia, Medicare compulsory insurance in 1972 brought equitable distribution of health care access, and Somkotra) reported a similar pro-poor impact of universal health insurance coverage in Thailand after 2005 [26,34]. With the introduction of free health care, England moved from a pro-rich to pro-poor health access [35]. With China’s continuing reform of health insurance, we anticipate a narrowing, but not closing, the need-based equal access gap.

Given the different coverage and deductible amounts for those with insurance, a large proportion of medical expense had to be met from individual or family resources. Our data show that the mean premium in rural Gansu was only 13RMB and 28RMB in rural Zhejiang. These are remarkably low premiums even compared to Gansu’s average rural incomes of 5000RMB. Low premiums mean a large gap between medical costs and out-of-pocket expenses. Equally telling is our survey evidence that in even in developed Zhejiang, about 20 percent of respondents reported they did not pay any NCMS, which meant that it was the poorest families and individuals who were not covered by any insurance. For the URBM scheme, premiums were much higher, around 300RMB per year in urban Gansu, and about the same for women in urban Zhejiang, but much higher, 600RMB per year, for males in urban Zhejiang. These high and differential premiums discriminated against the poor, contributing to the pro-rich bias in our results. These differentials in fees reflect the autonomy of county-level authorities to manage the insurance system and provide inpatient and outpatient care facilities.

In less-develop Gansu, fewer poor families and individuals belong to insurance schemes than in Zhejiang, and those that did belong to an insurance scheme in Gansu had less scope to finance out-of-pocket expenses, including for drugs that comprised as much of 40 percent of health budgets, compared to the richer population in Zhejiang [36]. These differences in health service availability and differential coverage meant the pro-poor impact of health insurance reform was blunted, except NCMS in developed Zhejiang. We speculate that the pro-poor bias in universal insurance schemes depended on some threshold level of provincial economic development and per capita income. Developed Zhejiang had reached the level of per capita income where poor rural individuals could supplement the insurance scheme through private contributions. But in cases where premiums were high and the level of per capita income low, the poor were disadvantaged in using health services, as occurred in Gansu and also urban Zhejiang. Urban poverty was as great an obstacle to accessing health services as was rural poverty. Of course, our inequality decomposition presents a descriptive analysis, indicating which variable contributes most to health service utilization inequality, rather than casual inferences.

Government intervention is crucial on three levels. First, Government needs to improve the provision of health care facilities. Health care facilities were not always available, even for patients able to pay. Second, Governments at all levels should standardize reimbursements to reduce the gap between urban and rural areas. This would reduce out-of-pocket medical expenses and redress high hospital expenses that prohibit members of poor families from utilizing health facilities. For example, the Government might implement an effective transfer payment mechanism, such as imposing a progressive tax or increasing the subsidy, for households enjoying the minimum living guarantee. While medical insurance should be mandatory, the policy premium should show preference to the poorest residents. Government policy directed to health insurance provision and coverage is moving in the right direction, with increased government investment and reform in health care.

Third, these policy changes are likely to be contingent on across-the-board economic growth coupled with income redistributive measures to ensure that the poor can financially access health care. The challenge is not simply to raise per capita income, but to reduce income disparity.

Our study focused on middle aged and old people. We found the middle and older aged poor to be concentrated geographically and by job. With age comes physical health deterioration. The health-age nexus mediated by job and geography means that with an aging population more rural and urban workers will seek medical care, particularly for non-communicable diseases. Both poor rural and urban workers face decreasing employment opportunities and increasing need for health care provision due to years of strenuous work in frequently unhealthy work environments.

There are several limitations to this study. All self-reported health status studies suffer from the respondents’ health consciousness level and health knowledge level [32]. Poor people might over or under report their actual health status due to inadequate knowledge, which can lead to an under or overestimation of the equity in health care utilization. This is the first major cross-provincial study on middle-aged and elderly access to health care, which needs to be repeated on larger data sets and data from other provinces.

The challenge to China of an aging population magnifies the challenge of ensuring that growth is equitable. China has reduced poverty, but both regional growth differentials and income inequality has increased significantly. Our data highlight the differences in health care access within the two provinces. Income was the most important factor in outpatient care in both provinces, but access to inpatient care was driven by a mix of income, need and non-need factors that significantly differed across and within the two provinces. These differences were the result of different levels of health care provision, different out-of-pocket expenses for health care and different access to and coverage of health insurance for rural and urban families. We argued that equity in health care utilization would not come simply by rising per capita incomes, but from addressing rising inequality in income distribution. Our findings are echoed in the World Bank China 2030 Report [4]:

"Reversing this trend [in rising inequality] requires three coordinated actions: delivering more and better quality public services to underserved rural areas and migrant populations… from primary health care to care for the aged; restructuring social security systems to ensure secure social safety…Mobilizing all segments of society…to share responsibilities in financing, delivering and monitoring the delivery of social services…[4]."

## Competing interests

The authors declare that they have no competing interests.

## Authors’ contributions

YW and JW conceptualized and supervised the study, contributed to the study design, made substantial contributions to the acquisition and quality assurance of the data, and analyzed the data. YZ takes a leading role in the acknowledge funding project in which makes it able to write the paper. EM and SN contributed to the study design, survey conduction and supervision, as well as interpretation and writing of the manuscript. ML contributed to the statistical analysis, interpretation, writing and finalizing of the manuscript. All authors read and approved the final manuscript.

## Pre-publication history

The pre-publication history for this paper can be accessed here:

http://www.biomedcentral.com/1472-6963/12/302/prepub
